# Detection of *14-3-3 sigma* (σ) promoter methylation as a noninvasive biomarker using blood samples for breast cancer diagnosis

**DOI:** 10.18632/oncotarget.13992

**Published:** 2016-12-16

**Authors:** Meng Ye, Tao Huang, Ying Ying, Jinyun Li, Ping Yang, Chao Ni, Chongchang Zhou, Si Chen

**Affiliations:** ^1^ The Affiliated Hospital of Ningbo University, Ningbo, Zhejiang 315020, People's Republic of China; ^2^ Zhejiang Provincial Key Laboratory of Pathophysiology, School of Medicine, Ningbo University, Ningbo, Zhejiang 315211, People's Republic of China; ^3^ Ningbo No. 2 Hospital, Ningbo, Zhejiang 315010, People's Republic of China

**Keywords:** 14-3-3 σ, promoter methylation, breast cancer, blood, diagnosis, biomarker

## Abstract

As a tumor suppressor gene, *14-3-3 σ* has been reported to be frequently methylated in breast cancer. However, the clinical effect of *14-3-3 σ* promoter methylation remains to be verified. This study was performed to assess the clinicopathological significance and diagnostic value of *14-3-3 σ* promoter methylation in breast cancer. *14-3-3 σ* promoter methylation was found to be notably higher in breast cancer than in benign lesions and normal breast tissue samples. We did not observe that *14-3-3 σ* promoter methylation was linked to the age status, tumor grade, clinic stage, lymph node status, histological subtype, ER status, PR status, HER2 status, or overall survival of patients with breast cancer. The combined sensitivity, specificity, AUC (area under the curve), positive likelihood ratios (PLR), negative likelihood ratios (NLR), diagnostic odds ratio (DOR), and pos*t-test* probability values (if the pretest probability was 30%) of *14-3-3 σ* promoter methylation in blood samples of breast cancer patients vs. healthy subjects were 0.69, 0.99, 0.86, 95, 0.31, 302, and 98%, respectively. Our findings suggest that *14-3-3 σ* promoter methylation may be associated with the carcinogenesis of breast cancer and that the use of *14-3-3 σ* promoter methylation might represent a useful blood-based biomarker for the clinical diagnosis of breast cancer.

## INTRODUCTION

Worldwide, breast carcinoma is the most common human malignancy and is the leading cause of cancer-related deaths among women. Approximately 1,676,600 new cases with breast cancer were clinically diagnosed, with an estimated 521,900 deaths occurring in 2012 [1]. Early detection using mammography screening is effective and can decrease mortality from this disease by up to 30% [2]. However, false positive mammograms can result in the over-diagnosis and over-treatment of breast carcinoma with tumor cell dissemination [3, 4]. Therefore, no novel biomarker has yet proven sufficiently sensitive or specific for the early detection and diagnosis of breast cancer in clinical practice.

Epigenetic alterations have been shown to be an early biotic event in human cancers [5, 6]. DNA methylation is a major form of epigenetic modification associated with target gene silencing and is correlated with cancer carcinogenesis and progression [5, 7, 8]. Some genes with aberrant promoter methylation have been identified as prognostic biomarkers in breast cancer (i.e., *RASSF1A*, *BRCA1* and *PITX2*) [9–11]. *14-3-3 σ*, a key member of the 14-3-3 protein family, promotes G2/M arrest and inhibits mitotic death [12]. As a tumor suppressor gene, *14-3-3 σ* has been reported to be downregulated in response to DNA methylation in a wide variety of human carcinomas, such as melanoma and ovary, prostate and endometrial carcinomas [13–15]. Studies have shown that *14-3-3 σ* is frequently inactivated by promoter methylation in breast cancer [16, 17]. Additionally, DNA methylation has been used as a noninvasive biomarker for cancer detection and diagnosis [18, 19]. *14-3-3 σ* promoter methylation in blood samples is a potential tool for the diagnosis of breast cancer [20, 21].

However, several studies have yielded controversial results with regard to the methylation frequency of *14-3-3 σ* promoter. Jeronimo *et al*. recorded that the *14-3-3 σ* promoter was methylated at the same rate in breast cancer patients and normal breast tissue samples [22]. Jing *et al*. reported a significant difference in the frequency of *14-3-3 σ* promoter methylation between breast cancer patients and cancer-free controls [23]. Therefore, we carried out this study to evaluate the relationship of *14-3-3 σ* promoter methylation with clinicopathological characteristics, and the prognostic role of *14-3-3 σ* promoter methylation in relation to overall survival (OS) or disease-free survival (DFS). In addition, we evaluated the diagnostic value of the *14-3-3 σ* promoter methylation test based on blood samples in breast cancer.

## RESULTS

### Characteristics of the included studies

**Figure 1:**
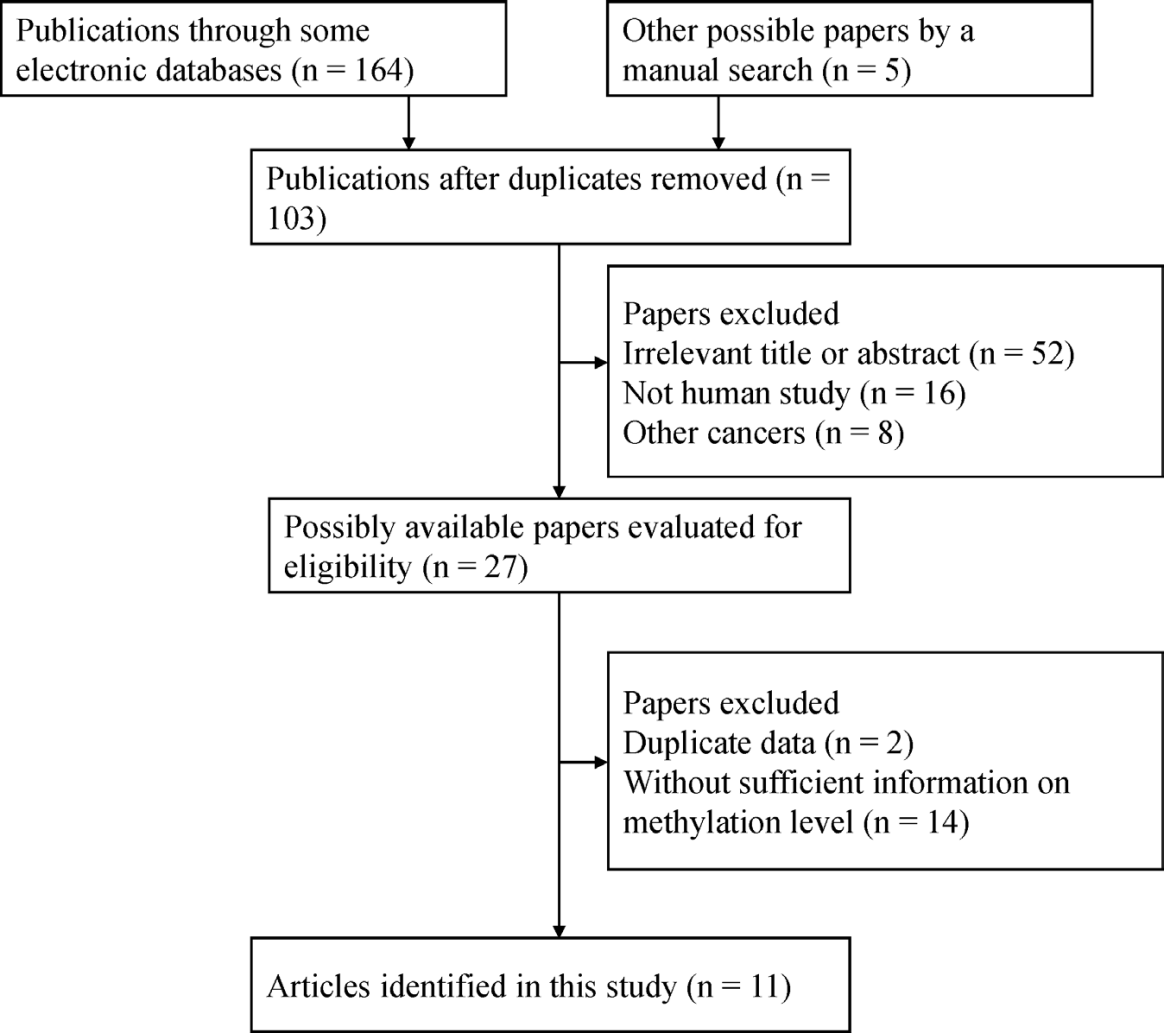
Flow chart of the selection process of the included studies

The selection process used for the potential studies is shown in Figure 1. According to the above inclusion criteria, we identified eleven case-control studies including a total of 2012 samples in this study [16, 17, 20–28]. Seven studies involving 417 breast cancer and 93 normal tissue samples were studied to analyze the correlation between *14-3-3 σ* promoter methylation and breast cancer [16, 17, 21–23, 25, 28]. Five studies involving 483 breast cancer and 301 benign lesions were examined to evaluate the relationship between *14-3-3 σ* promoter methylation and breast cancer [16, 20, 22, 23, 28]. Six studies including 646 breast cancer and 555 normal blood samples were studied to analyze the relationship between *14-3-3 σ* promoter methylation and breast cancer [20, 21, 23, 24, 26, 27]. Four studies were examined to assess the association between *14-3-3 σ* promoter methylation and clinicopathological characteristics [16, 21, 23, 27]. Two studies with 170 breast cancer patients reporting original data on OS were examined using univariate analysis [16, 27]. The general characteristics of the included studies are listed in Table 1.

**Table 1:** General characteristics of the included studies

First author	Country	Ethnicity	Age	Stage	Method	Sample	Cancer	Benign lesions	Normal	OS	Expression
							*N* (M %)	*N* (M %)	*N* (M %)		
Ferguson 2000 [17]	USA	European	NA	NA	MSP	Tissue	82 (91.5)		6 (0)	NA	Negative
Umbricht 2001 [28]	USA	European	NA	NA	MSP	Tissue	43 (90.7)	8 (37.5)	10 (0)	NA	ND
Jing 2007 [23]	China	Asian	32–73	0–3	MSP	Tissue	38 (86.8)	20 (0)	20 (0)	NA	ND
Jing 2007 [23]	China	Asian	32–73	0–3	MSP	Blood	38 (55.3)		50 (0)	NA	ND
Jeronimo 2008 [22]	Portugal	European	63	1–4	qMSP	Tissue	66 (100)	24 (79.2)	12 (100)	NA	ND
Jing 2008 [27]	China	Asian	49.1	NA	MSP	Blood	102 (82.4)		20 (0)	NS	ND
Luo 2010 [16]	China	Asian	33–74	NA	MSP	Tissue	68 (89.7)	13 (15.4)	10 (0)	NS	Negative
Mirza 2010 [21]	India	European	50	1–3	MSP	Tissue	100 (61)		15 (20)	NA	NS
Mirza 2010 [21]	India	European	50	1–3	MSP	Blood	100 (56)		30 (6.7)	NA	ND
Gheibi 2012 [25]	Iran	European	51.7	NA	MSP	Tissue	20 (70)		20 (20)	NA	ND
Sharma 2012 [26]	India	European	32–76	NA	MSP	Blood	30 (83.3)		30 (0)	NA	ND
Wang 2014 [24]	China	Asian	56.2	NA	MSP	Blood	108 (58.3)		180 (10)	NA	ND
Shan 2016 [20]	China	Asian	NA	NA	qMSP	Blood	268 (73.5)	236 (61)	245 (58.4)	NA	ND

MSP: methylation-specific polymerase chain reaction; qMSP: quantitative methylation-specific polymerase chain reaction; M: methylation; N: sample size, NA: not applicable; NS: not significant; ND: not done; OS: overall survival.

### Association between *14-3-3 σ* promoter methylation and breast cancer in cancer vs. controls

**Figure 2:**
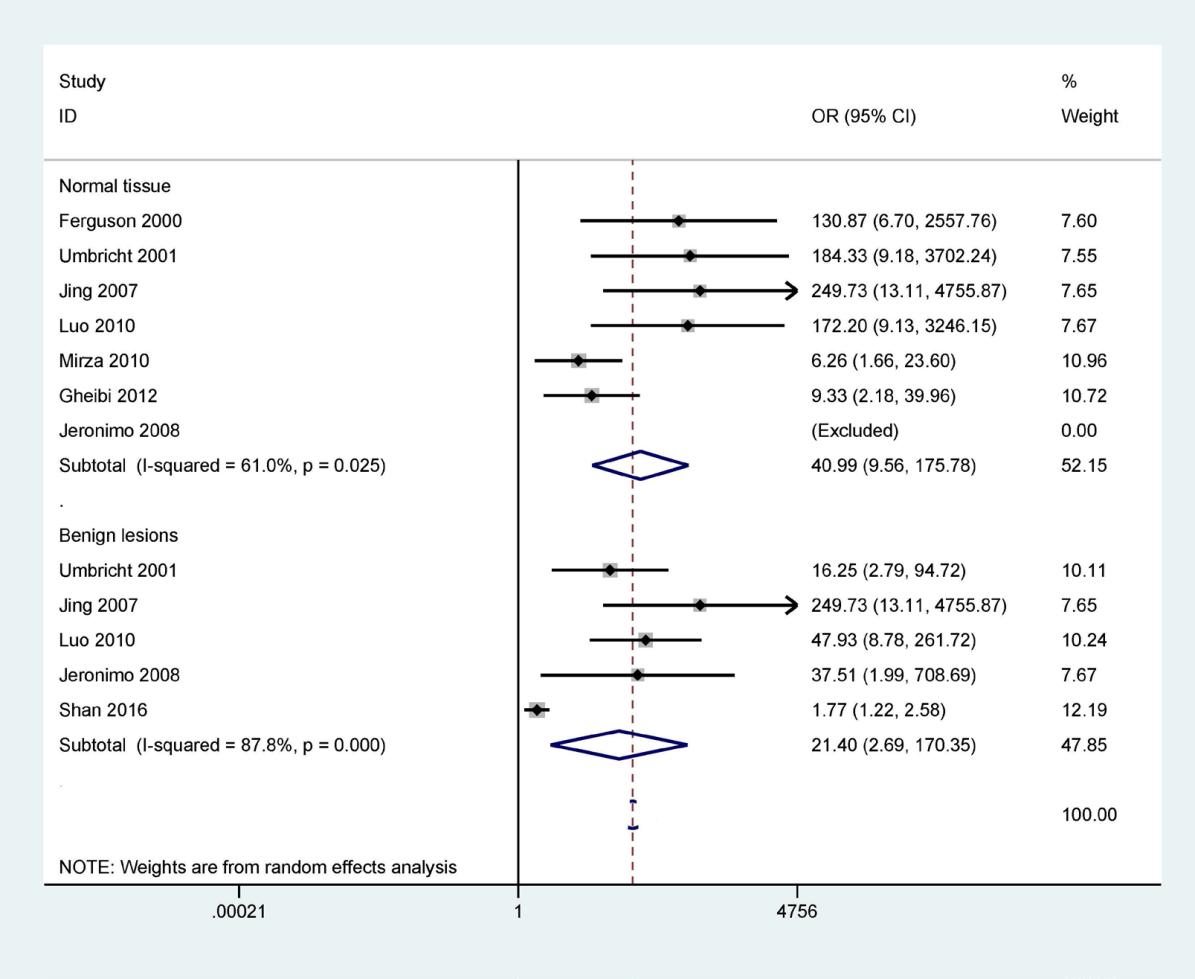
Forest plot of 14-3-3 σ promoter methylation and breast cancer indicating the combined OR including 7 studies with 417 breast cancer and 93 normal tissue samples and 5 studies with 483 breast cancer and 301 benign lesions; cancer vs. benign lesions: OR = 21.40, 95% CI = 2.69–170.35, P = 0.004; cancer vs. normal breast tissues: OR = 40.99, 95% CI = 9.56–175.78, P < 0.001

Figure 2 shows that the methylation frequency of the *14-3-3 σ* promoter is significantly higher in breast cancer than in benign lesions and normal breast tissues (OR = 21.40, 95% CI = 2.69–170.35, *P* = 0.004; OR = 40.99, 95% CI = 9.56–175.78, *P* < 0.001), indicating that *14-3-3 σ* promoter methylation is significantly associated with an increased risk of breast cancer.

**Figure 3:**
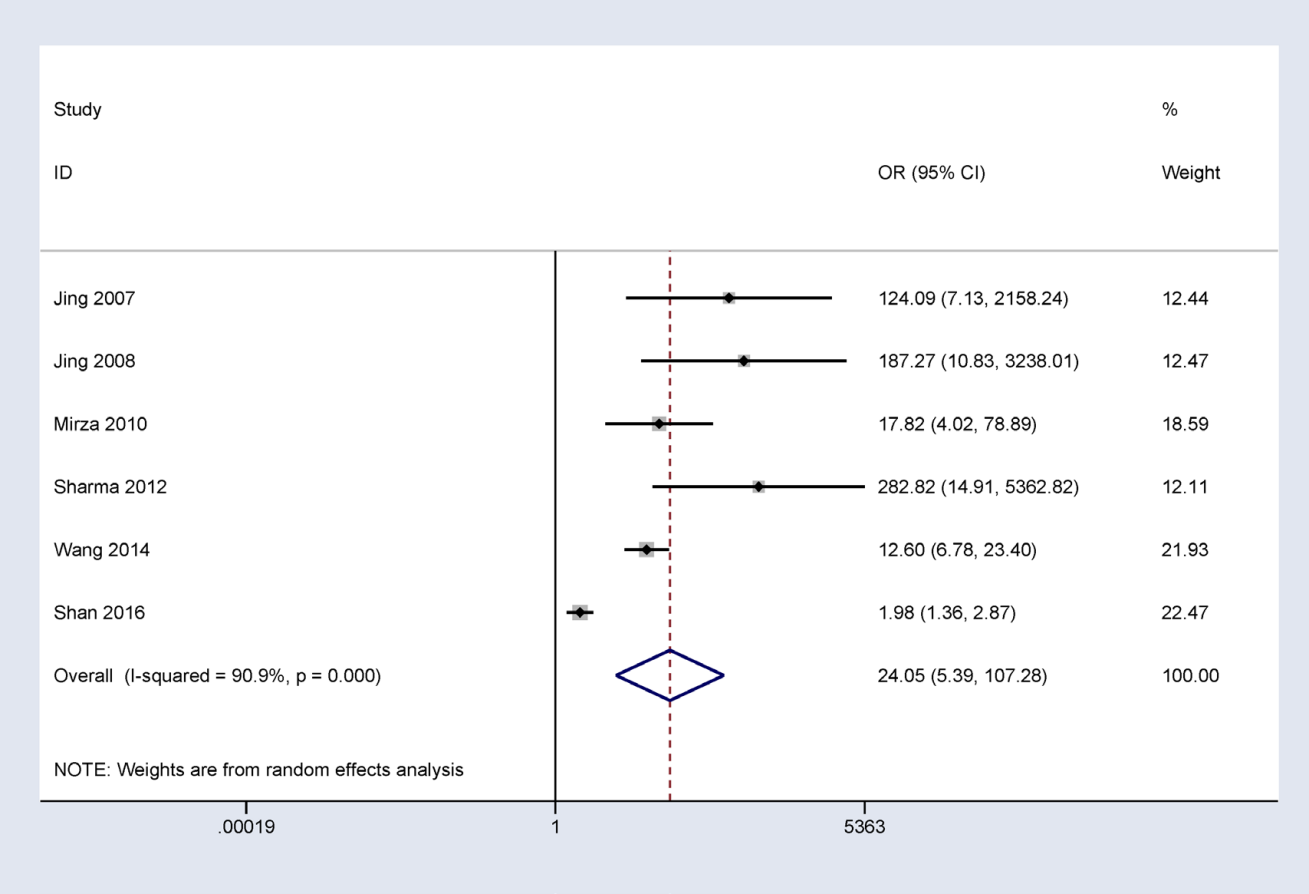
Forest plot of 14-3-3 σ promoter methylation and breast cancer indicating the combined OR of blood samples involving 6 studies with 646 breast cancer and 555 normal blood samples from healthy subjects; OR = 24.05, 95% CI = 5.39–107.28, P < 0.001

In addition, when normal and cancer-related blood samples were compared, a significant correlation was also observed between *14-3-3 σ* promoter methylation and breast cancer in blood samples (OR = 24.05, 95% CI = 5.39–107.28, *P* < 0.001) (Figure 3).

### Subgroup analysis by ethnicity in cancer vs. controls

**Figure 4:**
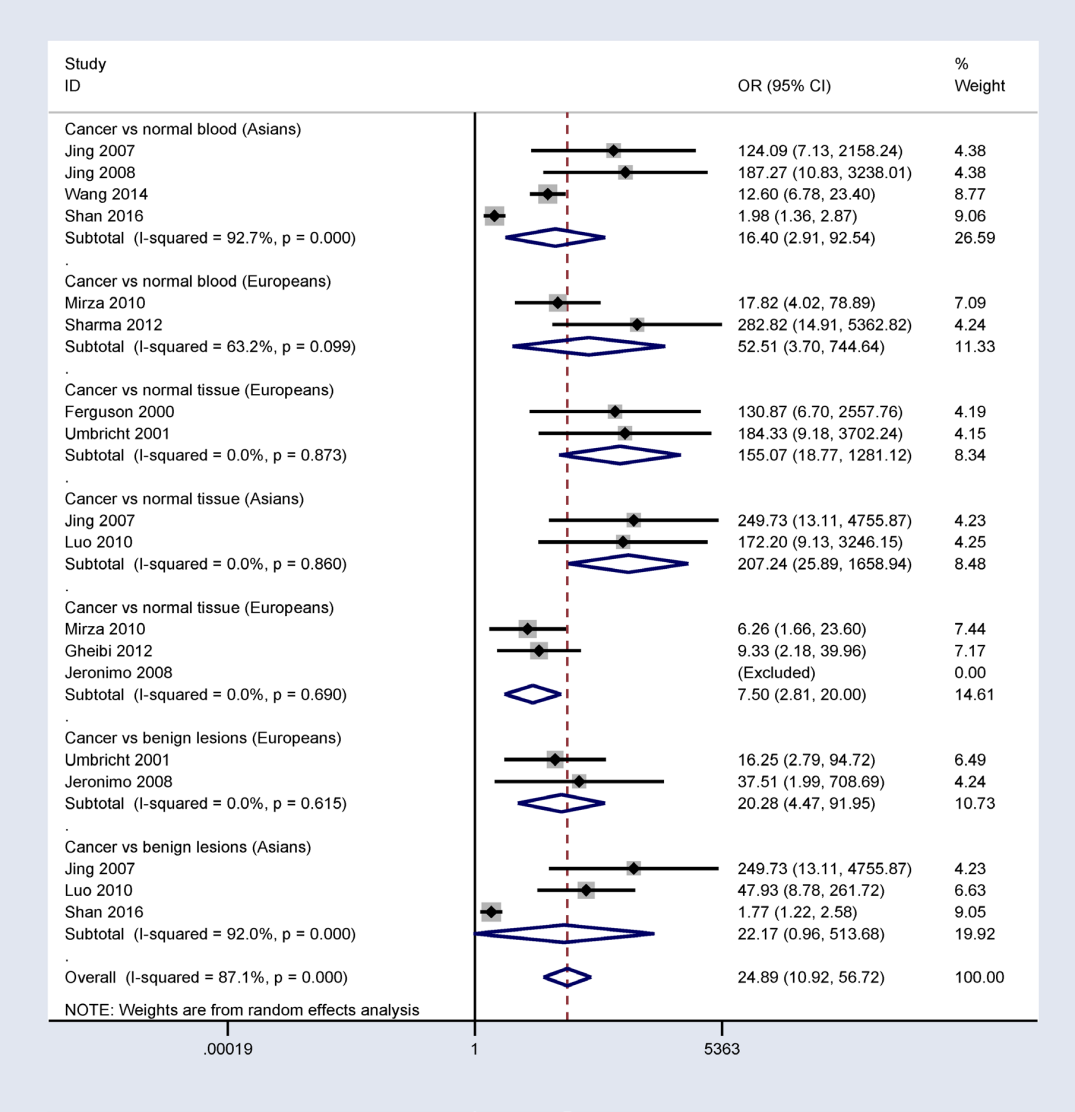
Subgroup analysis by ethnicity in patients with breast cancer vs. controls

A subgroup analysis based on ethnicity (Asian and European populations) showed that *14-3-3 σ* promoter methylation was correlated with breast cancer risk in Asian and European populations (Figure 4).

### Association of *14-3-3 σ* promoter methylation with clinicopathological features

**Figure 5:**
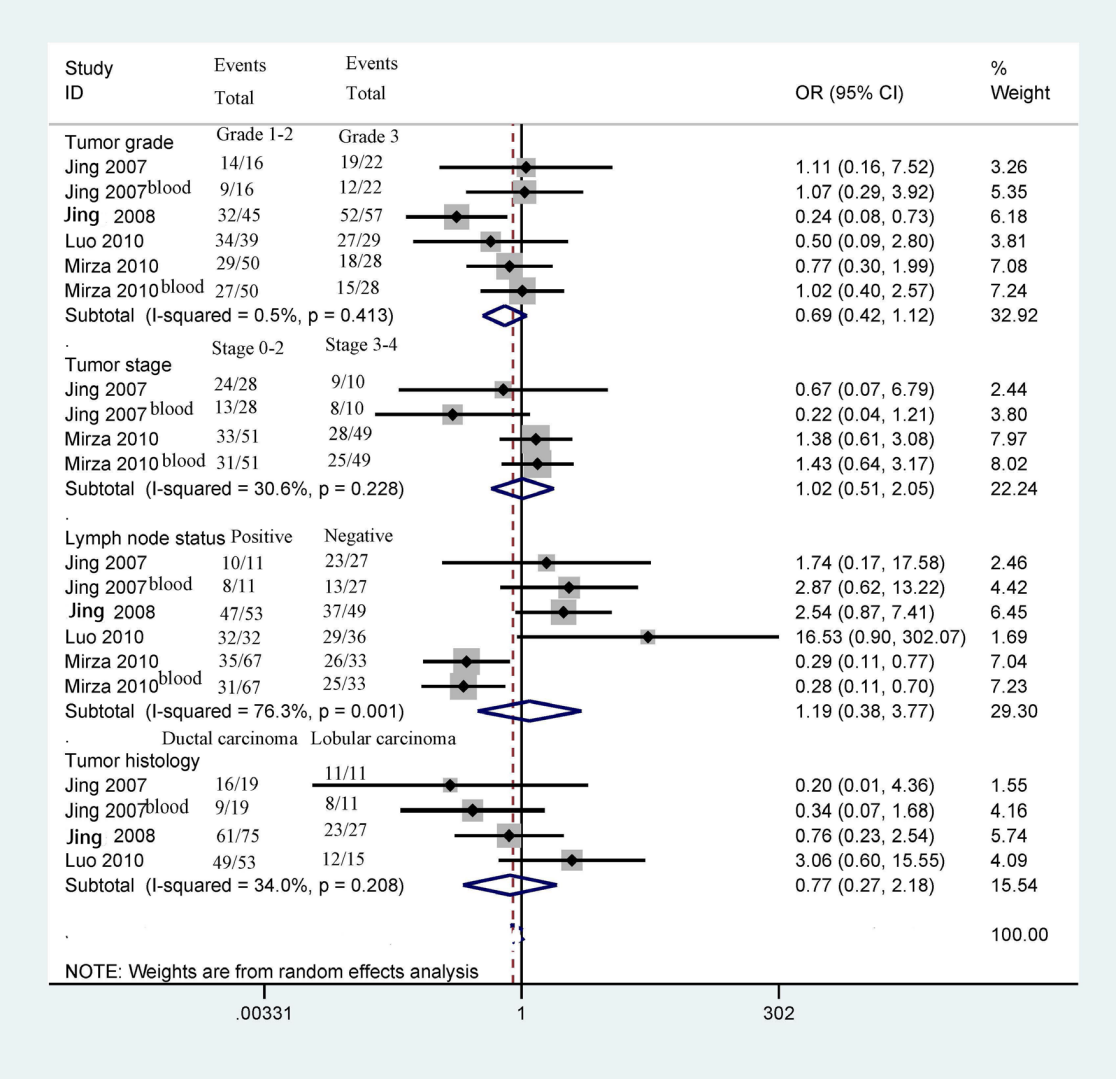
Forest plot of *14-3-3 σ* promoter methylation and some clinicopathological features demonstrating the pooled OR from 4 studies, such as tumor grade, clinical stage, lymph node status, and tumor histology (all *P* > 0.1)

**Figure 6:**
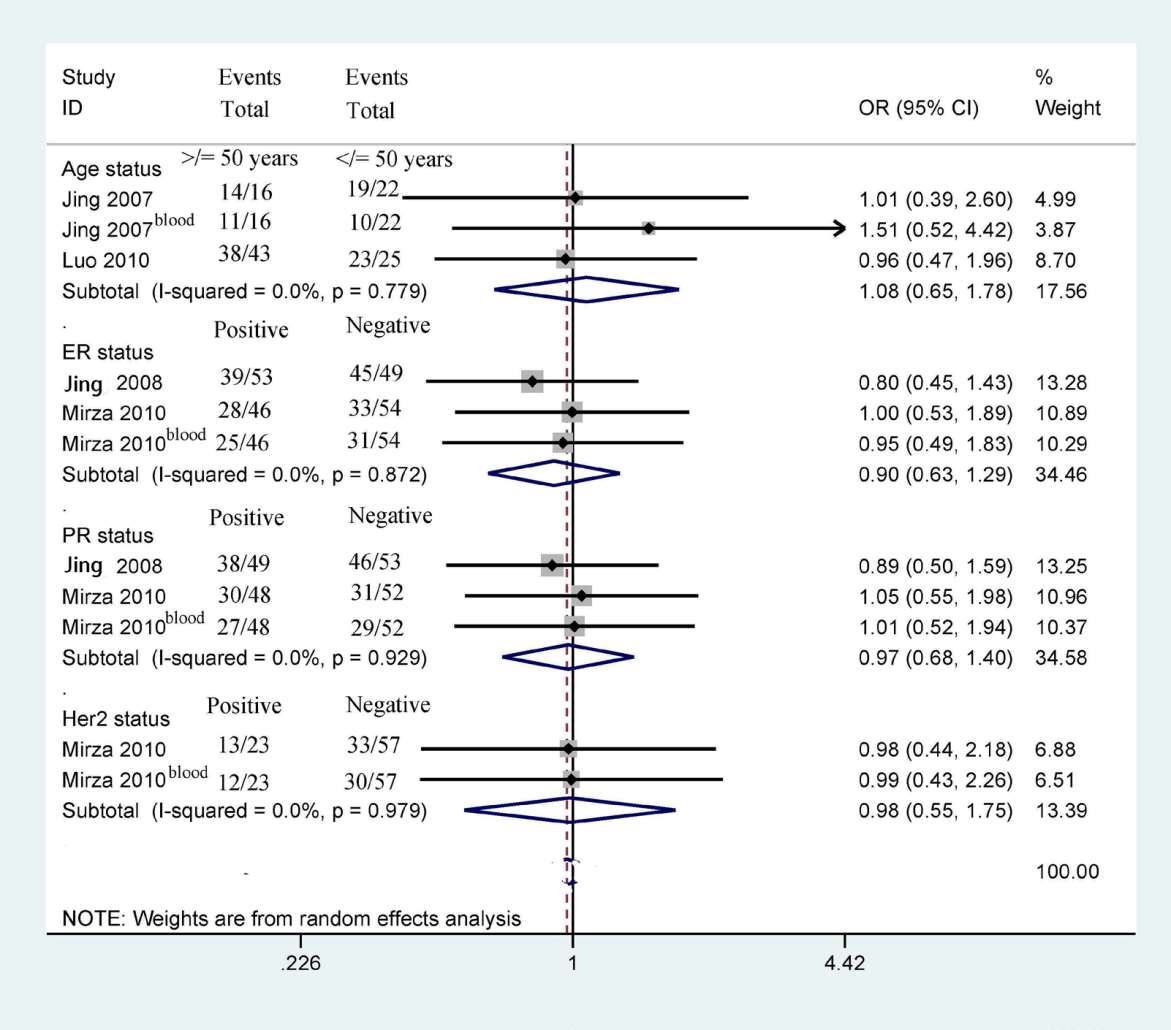
Forest plot of *14-3-3 σ* promoter methylation and other clinicopathological features demonstrating the pooled OR from 4 studies, such as age status, ER status, PR status, and Her2 status (all *P* > 0.1)

Next, we determined whether *14-3-3 σ* promoter methylation was correlated with clinicopathological characteristics in breast cancer, including age status, tumor grade, clinical stage, lymph node status, histological subtype, ER status, PR status, and HER2 status (Figures 5 and 6). Our findings demonstrated that *14-3-3 σ* promoter methylation was not associated with clinicopathological characteristics (all *P* > 0.1). However, analyses of *14-3-3 σ* promoter methylation with clinicopathological characteristics should be carefully considered because the sample sizes examined in our study were small.

### Prognostic value of *14-3-3 σ* promoter methylation in patients with breast cancer

Only two studies, involving 170 breast cancer patients, reported that *14-3-3 σ* promoter methylation was not significantly linked to the prognosis of breast cancer patients in OS using univariate analysis [16, 27]. Additional studies with large sample sizes are needed to examine DFS and OS using multivariate analysis.

### Sensitivity analyses of *14-3-3 σ* promoter methylation

**Figure 7:**
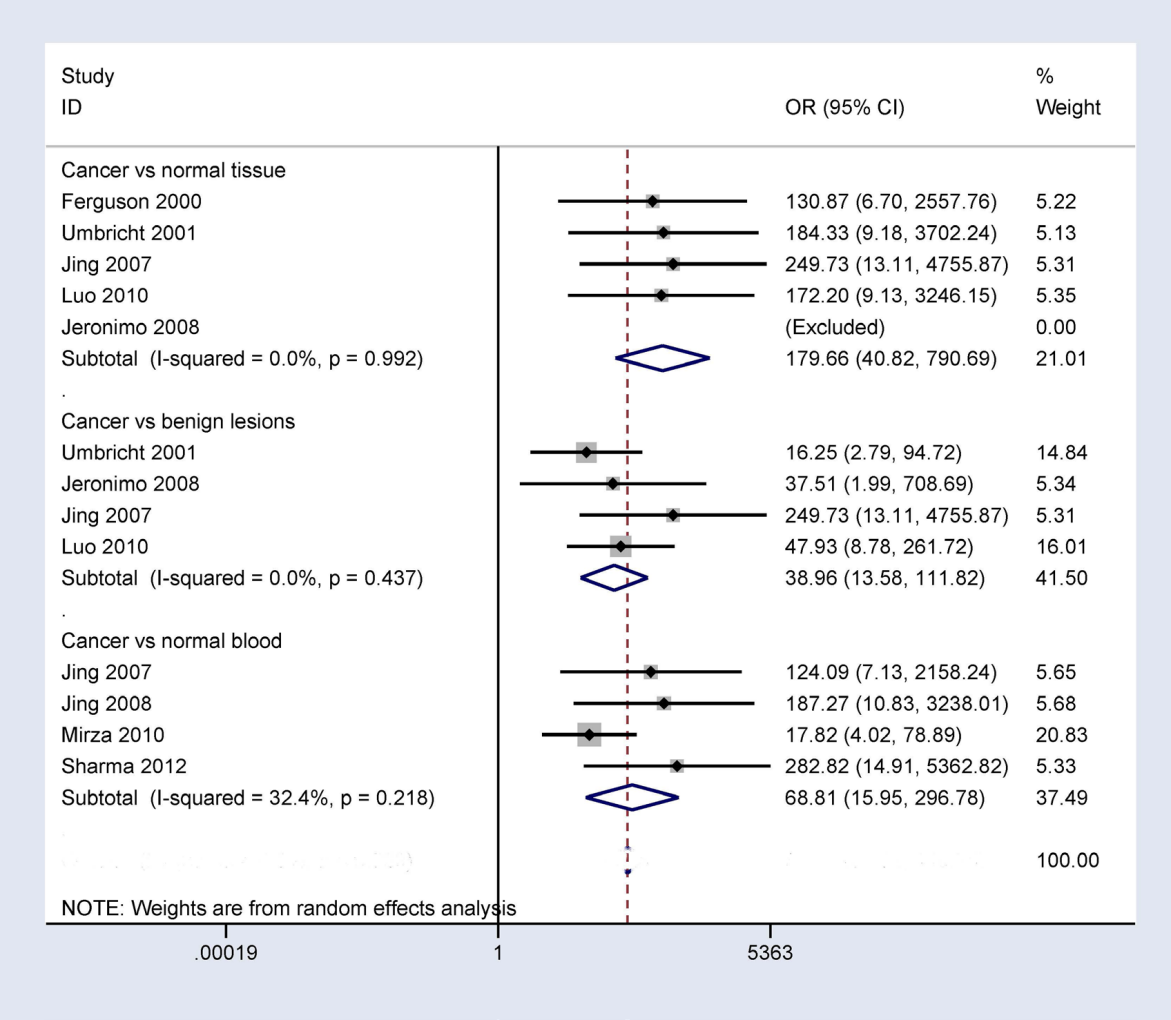
Forest plot of *14-3-3 σ* promoter methylation and breast cancer based on sensitivity analyses performed by omitting one individual study in breast cancer vs. benign lesions, normal breast tissues, and normal blood samples

When breast cancer was compared to benign lesions, normal tissues, and normal blood samples, substantial heterogeneity was measured in this study (*I^2^* = 87.8%, *P* < 0.001; *I^2^* = 61.0%, *P* = 0.025; and *I^2^* = 90.9%, *P* < 0.001, respectively). We conducted sensitivity analyses to estimate the stability of the overall OR and changes in heterogeneity by omitting an individual study. As shown in Figure 7, in the comparison of breast cancer and benign lesions, one study [20] was deleted, and the pooled OR from the remaining studies was re-calculated (OR = 38.96, 95% CI = 13.58–111.82), resulting in decreased heterogeneity (*I^2^* = 0.0%, *P* = 0.437). When comparing breast cancer and normal tissue samples, two studies [21, 25] were successively removed, and the combined OR from the remaining studies was re-calculated (OR = 179.66, 95% CI = 40.82–790.69), resulting in no obvious evidence of heterogeneity (*I^2^* = 0.0%, *P* = 0.992). When comparing breast cancer and normal blood samples, we deleted two studies [20, 24] and re-calculated the pooled OR (OR = 68.81, 95% CI = 15.95–296.78), yielding no significant heterogeneity (*I^2^* = 32.4%, *P* = 0.218). Our analyses showed that the results of the overall OR remained significant, indicating that our results were stable and authentic.

### Diagnostic value of **14-3-3 σ** promoter methylation using blood testing

**Figure 8:**
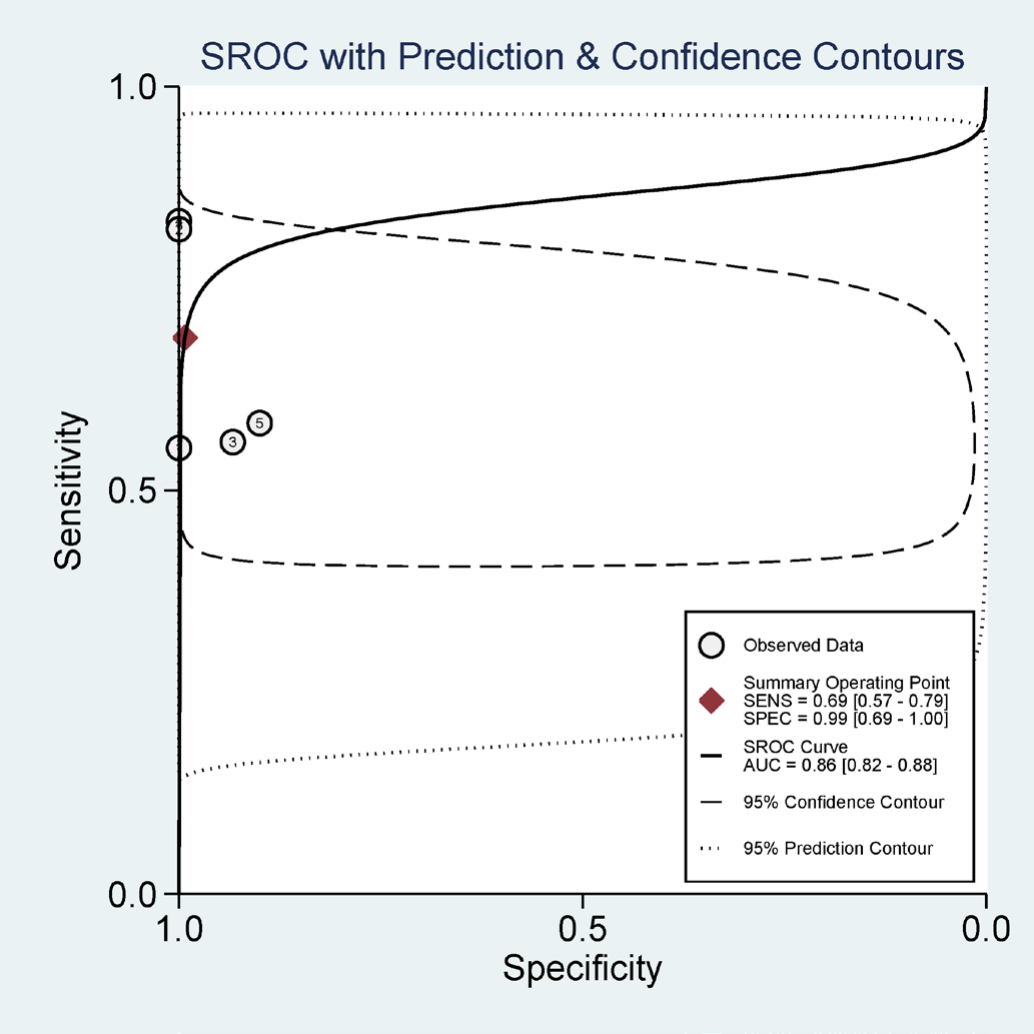
Summary receiver operating characteristics (SROC) estimation for the diagnostic value of *14-3-3 σ* promoter methylation based on blood samples in breast cancer vs. healthy subjects; sensitivity = 0.69, specificity = 0.99, and the area under the curve (AUC) = 0.86

**Figure 9:**
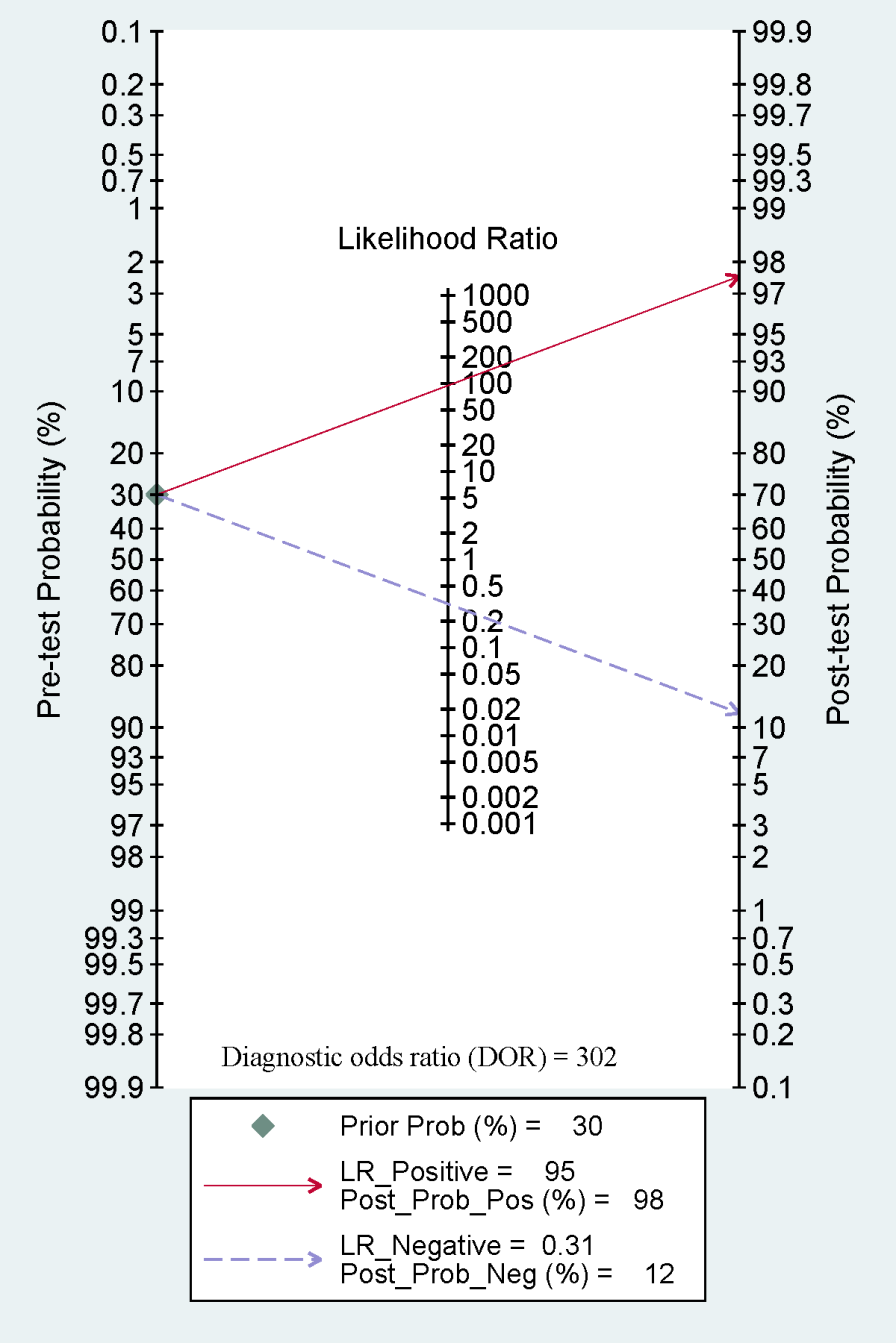
A Fagan nomogram showing pooled positive likelihood ratios (PLR) of 95, negative likelihood ratios (NLR) of 0.31, a diagnostic odds ratio (DOR) of 302, and post-test probability values of 98% (if pretest probability was 30%)

Furthermore, we calculated the pooled sensitivity, specificity, AUC, PLR, NLR, DOR, and post-test probability values from five studies using the MSP method to evaluate the diagnostic value of **14-3-3 σ** promoter methylation for breast cancer diagnosis in blood samples. The combined sensitivity, specificity and AUC of **14-3-3 σ** promoter methylation using MSP detection were 0.69 (95% CI: 0.57–0.79), 0.99 (95% CI: 0.69–1.00), and 0.86 (95% CI: 0.82–0.88), respectively (Figure 8). The pooled PLR, NLR, DOR, and post-test probability values (if the pretest probability was 30%) were 95, 0.31, 302, and 98%, respectively (Figure 9). In this study, the pre-test probability value obtained from the Fagan nomogram was defined as 30%, with a positive LR value of 95. The positive post-test probability value was 98%, and the negative LR value was 0.31. The negative post-test probability value was 12%. This result can be described as follows: if a patient had a 30% possibility of breast cancer based on early detection, there was a possibility of 98% that a definitive diagnosis of breast cancer would be made if the **14-3-3 σ** promoter methylation detection result was positive. When the test was negative, the patient had a 12% possibility of having breast cancer. Thus, **14-3-3 σ** promoter methylation testing using the MSP method will increase the positive diagnosis rate by 68% and the negative rate by 18%. In addition, we found that the mean **14-3-3 σ** promoter methylation rate was 0.66 in breast cancer and 0.06 in blood samples obtained from healthy subjects. Therefore, we showed that **14-3-3 σ** promoter methylation using non-invasive blood detection can guide the diagnosis of breast cancer in clinical applications. Larger studies are warranted to verify these findings.

## DISCUSSION

Breast cancer is a heterogeneous and hormone-dependent disease and is mainly attributed to the promoter methylation of tumor suppressor genes (TSGs) involving cell proliferation, cell death, cell migration, and cell invasion, all of which lead to the development of this disease [29]. The absence or downregulation of gene expression, which is correlated with CpG islands related to the promoter methylation of different genes, has been shown in many cancers, including breast cancer [30–32]. Increasing evidence has demonstrated that **14-3-3 σ** promoter methylation is involved in the pathogenesis and development of several carcinomas, including hepatocellular carcinoma [33], nasopharyngeal carcinoma [34], and breast cancer [16]. Some studies have shown a significant association between **14-3-3 σ** promoter methylation and its expression in breast cancer with the absence of **14-3-3 σ** expression [16, 17]. The site of **14-3-3 σ** promoter methylation is located after the transcription initiation site and before the downstream promoter region. Although previous studies have evaluated the frequency of **14-3-3 σ** promoter methylation in malignant and cancer-free breast tissue samples, the role of **14-3-3 σ** promoter methylation in breast cancer remains controversial. Thus, this study was performed to achieve further insight into the role of **14-3-3 σ** promoter methylation in breast cancer carcinogenesis, progression, and prognosis.

Jeronimo *et al*. reported that **14-3-3 σ** promoter methylation is high in breast cancer, benign lesions, and normal tissue samples, with no significant association [22]. Jing *et al*. reported that **14-3-3 σ** promoter methylation was significantly higher in breast cancer than in benign lesions and normal tissues [23]. The results based on the combined OR showed that **14-3-3 σ** promoter methylation status was significantly higher in breast cancer tissues than in benign lesions and normal breast tissues, indicating that **14-3-3 σ** promoter methylation may be correlated with the carcinogenesis of breast cancer.

Our results showed no significant correlation between **14-3-3 σ** promoter methylation status and clinicopathological features, including age status, tumor grade, clinic stage, lymph node status, histological subtype, ER status, PR status, and HER2 status (all *P* > 0.1). Additionally, two studies involving 170 breast cancer patients reported that **14-3-3 σ** promoter methylation was not significantly associated with OS in breast cancer patients using univariate analysis [16, 27], suggesting that **14-3-3 σ** promoter methylation may not be correlated with the prognosis of patients with breast cancer in terms of OS. However, the above results regarding **14-3-3 σ** promoter methylation and its association with clinicopathological parameters and overall survival in breast cancer patients should be considered cautiously because the sample sizes analyzed in the present study were small.

The pooled OR showed a significant relationship between **14-3-3 σ** promoter methylation status and breast cancer in the blood, indicating that **14-3-3 σ** promoter methylation may be a useful biomarker when testing blood samples in relation to breast cancer. Some studies have suggested that DNA methylation testing in body fluid samples can yield a molecular biomarker for cancer screening and diagnosis [35, 36]. Hence, we further assessed the diagnostic effect of **14-3-3 σ** promoter methylation based on blood samples in patients with breast cancer vs. healthy subjects. Using MSP method, **14-3-3 σ** promoter methylation exhibited a pooled sensitivity of 0.69, a specificity of 0.99, an AUC of 0.86, and a DOR of 302, indicating that **14-3-3 σ** promoter methylation is a good biomarker for breast cancer diagnosis. Approximately 30% of women with breast cancer can be diagnosed at an early stage [37]. The pre-test probability value obtained from the Fagan nomogram was defined as 30%, and the pooled PLR, NLR, and post-test probability values were 95, 0.31, and 98%, respectively. We also found that the mean methylation of the **14-3-3 σ** promoter was very significantly higher in breast cancer samples than in blood samples obtained from healthy subjects (0.66 vs. 0.06, respectively). Although only two of the eligible studies of blood examined here, which were based on 79 early stage patients and 58 advanced stage patients, reported clinical stage information [21, 23], a similar methylation level was found during early stage and advanced stage breast cancer (0.56 vs. 0.57, respectively). In the clinical setting, carbohydrate antigen 153 (CA153), a more specific and sensitive marker than cancer embryonic antigen (CEA), may be applied as a noninvasive biomarker for breast screening, but not a very useful biomarker for breast cancer diagnosis because of a low sensitivity (sensitivity: ∼ 0.63; specificity: ∼0.82), especially early breast cancer [38–40]. The above analyses suggest that **14-3-3 σ** promoter methylation may be a promising blood-based biomarker for the early and non-invasive diagnosis of patients with breast cancer. More clinical research studies involving large sample sizes are necessary to validate our findings.

Significant heterogeneity was observed in breast cancer vs. control groups. We conducted sensitivity analyses to evaluate the stability of the pooled OR. One study [20] was deleted when comparing cancer vs. benign lesions, two studies [21, 25] were removed when comparing cancer vs. normal tissues, and two studies [20, 24] were deleted when comparing cancer vs. normal blood samples. The results showed that the pooled OR remained significant with no substantial heterogeneity, indicating that the analyses were stable. The results obtained regarding observed bias in our study were not very clear, possibly due to the use of inappropriate or different primers and conditions for the detection of **14-3-3 σ** promoter methylation.

Several limitations existed in this study. First, papers published in English were included in this study; the exclusion of studies written in other languages might have led to selection bias. Second, because the studies on the association between **14-3-3 σ** promoter methylation and clinicopathological parameters and overall survival were based on very small sample populations, further studies with larger sample populations are needed. Third, the included studies were case-control design, but not prospective design. Moreover, eligible studies were lack of the detailed stage of patients with breast cancer. Based on the reporting recommendations for tumor marker prognostic studies (REMARK) [41], further multi-center and well-matched prospective studies with large sample sizes (early stage or advanced stage) are very necessary to validate the diagnostic capacity of **14-3-3 σ** promoter methylation in breast cancer in the future. Fourth, additional studies using quantitative methods such as quantitative MSP (QMSP) and pyrosequencing are essential. Finally, the detection of methylated *SEPT9* DNA was recommended as a noninvasive marker-based blood test for colorectal cancer screening in April 2016 [42], which suggests that gene methylation has a potential value as a noninvasive biomarker for cancer screening and diagnosis.

In conclusion, our findings show that **14-3-3 σ** promoter methylation status may be correlated with an increased risk of breast cancer. **14-3-3 σ** promoter methylation is not associated with the clinicopathological parameters and overall survival of patients with breast cancer. **14-3-3 σ** promoter methylation might be a useful diagnostic biomarker for the clinical diagnosis of breast cancer based on blood samples. Further large-scale studies are necessary to further study the role of **14-3-3 σ** promoter methylation in relation to the prognosis and clinical management of patients with breast cancer.

## MATERIALS AND METHODS

### Literature search

A range of digital databases including the PubMed, Embase, EBSCO and the Cochrane Library were comprehensively searched to identify potential articles that were published prior to September 10th, 2016. The key words and search terms used were as follows: (14-3-3* OR SFN OR stratifin) AND (mammary OR breast) AND (cancer OR tumor OR neoplasm OR carcinoma) AND (methylation OR epigene*). To obtain other potentially relevant studies, reference lists from the included articles were also carefully scanned.

### Eligibility criteria

The following selection criteria for eligible studies were used in this study: 1) the patients were limited to those with primary breast cancer based on histopathology, without restriction regarding sample type; 2) case–control or cohort studies included sufficient data regarding the methylation frequency of the *14-3-3 sigma* (*σ*) promoter in breast cancer, benign lesions, and normal samples; 3) full-text articles published in English were selected in our study; 4) if authors published more than one publication using the same population or overlapping data, only the most complete study with the most data was selected. Papers that did not satisfy the above inclusion criteria were excluded. The detailed characteristics of the included studies were shown in Table 1.

### Data extraction

The data from the available studies were independently reviewed and extracted by two authors. Inconsistent information was discussed by all authors. Data recorded from the included studies included the last name of the first author, year of publication, detection method, country, ethnic population, tumor stage, sample type, expression information, methylation level, number of study subjects, number of methylations, overall survival (OS), disease-free survival (DFS), and clinicopathological characteristics such as age status, tumor grade, clinical stage, lymph node status, histological subtype, estrogen receptor (ER) status, progesterone receptor (PR) status and human epidermal growth factor receptor-2 (HER2) status. Normal blood samples were obtained from healthy subjects, and normal tissues were obtained from breast samples without tumor cells.

### Statistical analysis

The data were analyzed using Stata statistical software, version 12.0 (Stata Corp, College Station, TX, USA). The overall odds ratios (ORs) with 95% confidence intervals (CIs) were measured to evaluate the strength of the relationship between **14-3-3 σ** promoter methylation and breast cancer in cancer vs. benign lesions, normal samples, and clinicopathological features. Data on OS or DFS were extracted from the original studies and were recalculated based on the pooled hazard ratios (HRs) with their 95% confidence intervals (CIs) if possible. The Cochran's Q statistic and I^2^ tests were used to estimate potential heterogeneity [43]. The pooled results were calculated using the random-effects model. When significant heterogeneity among studies was observed in the current study (*I^2^* ≥ 50% or *P* < 0.1), we conducted sensitivity analyses to determine the influence and stability of an individual study on the pooled results based on the omission of a single study [44]. Pooled sensitivity and specificity were commonly used to estimate the diagnostic effect. However, according to the threshold effect, these two traditional values occasionally did not reflect the overall accuracy of the test. Thus, the summary receiver operator characteristic (SROC) curve was constructed, and the area under the SROC curve (AUC) value was used to explore the stability and accuracy of the diagnostic value in a meta-analysis [45, 46]. Fagan nomograms were also used as measures of pre-test probability and post-test probability values in clinical practice [47]. The pooled sensitivity, specificity, AUC, positive likelihood ratios (PLR), negative likelihood ratios (NLR), diagnostic odds ratio (DOR), and post-test probability values using a pre-test probability of 30% were applied and summarized to estimate the diagnostic value of **14-3-3 σ** promoter methylation using methylation-specific polymerase chain reaction (MSP) detection in blood samples.
